# Orthoptic stroke services in the UK and Ireland: how have they evolved?

**DOI:** 10.1038/s41433-026-04243-4

**Published:** 2026-01-17

**Authors:** Lauren R. Hepworth, Fiona J. Rowe

**Affiliations:** Institute of Population Health, University of Liverpool, Liverpool, UK

**Keywords:** Rehabilitation, Brain injuries

## Abstract

**Background:**

There are ~100,000 new strokes in the UK each year, 60% experience a new stroke-related visual impairment. Orthoptic stroke services were surveyed as baseline and follow-up in 2007 and 2017. Three new guidelines relating to post-stroke vision were published 2023–2025 and added to a mandatory national audit programme. This study aimed to assess the current provision of stroke orthoptic services since the 2016 National Clinical Guideline update through to 2023 updates.

**Methods:**

An online survey was circulated to registered orthoptists in the UK and Ireland.

**Results:**

A total of 206 responses representing 125 orthoptic departments (67% response rate) were received, from all five nations. Stroke vision services were reported to be provided by 66.1% of orthoptic departments in hospitals with a stroke unit, of which only 27.6% were funded. Services often rely on vision screening by the stroke team (84.2%) with referral to orthoptic services (65.8%), only half of which were reported to be using a validated screening tool. To cover a mean of 42.5 beds, a mean of 0.43 full-time equivalent orthoptic staffing dedicated to stroke care and 0.27 funded, with lower medians.

**Conclusions:**

Since 2017, stroke unit vision screening provision has increased, however over the same period a reduction occurred in funded services. Health inequality persists in that there remain areas with no/poor provision of post-stroke vision services. This study provides a baseline by which to assess change following the publication/updates of national and international guidelines and national audit programmes strengthening recommendations for post-stroke vision care.

## Introduction

Each year in the United Kingdom (UK), 100,000 individuals experience a stroke, with approximately 1.3 million individuals living in the UK with the effects of stroke [1]. The prevalence of visual impairment post-stroke is 73%, and an incidence of new stroke-related visual impairment being 60% [2]. Therefore, there is a considerable population living with visual impairment due to stroke. The numbers living with stroke globally is expected to increase due to aging populations and improved survival, with an expected 27% rise in the European Union by 2047.

The European Stroke Organisation (ESO) guideline on visual impairment in stroke recommends visual screening post-stroke but with a preference for specialist vision assessment to improve detection rates and accuracy of diagnosis [3]. There has been growing specialist vision assessment provision in the UK and Ireland over the past two decades where orthoptists are involved. An orthoptist is an allied health professional specialising in the assessment, diagnosis and management of eye movement abnormalities and related visual conditions. Orthoptists are listed as members of the stroke multi-disciplinary team in the National Clinical Guidelines for Stroke for the UK and Ireland [4, 5].

The first survey of UK orthoptic stroke services was conducted in 2007 and found that 45% of stroke services did not provide any formal vision assessment, and for those that did, this was limited to visual acuity only or the details not known in 15% [6]. Vision stroke services were led by orthoptists in 41% of services [6]. A second survey of orthoptic stroke services was conducted in the UK and Ireland in 2017 [7]. The results of this survey indicated an improvement in the provision of eye care services for stroke survivors since 2007, with 98% of respondents reporting provision of a stroke vision service. However, there remained a lack of orthoptists providing a service specifically on stroke units (48%) and vision care was being delivered in outpatient clinics for many stroke survivors [7]. Health inequalities have been identified by stroke survivors with visual impairment including a lack of integrated stroke and vision care resulting in a lack of information and early access to eye care [8].

Since the 2007 orthoptic survey, the National Clinical Guidelines for Stroke were updated in 2016. A major change for vision care was that orthoptists were added as members of the core acute stroke unit multidisciplinary team [5]. Both the National Clinical Guidelines for Stroke for the UK and Ireland and the National Institute for Health and Care Excellence (NICE—applicable to England and endorsed by Wales, Scotland and Northern Ireland) published further updated guidelines in 2023, and a dataset change to the Sentinel Stroke National Audit Programme (SSNAP) was implemented in October 2024 [4, 9, 10]. The changes to the National Clinical Guidelines for Stroke for the UK and Ireland included recommendations for orthoptic staffing levels, that screening for vision problems should be performed by an appropriate professional using a standardised approach and treatment programmes should be developed with an orthoptist [4]. The changes to the NICE guidelines for stroke rehabilitation in adults were more specific including recommending a specialist orthoptic assessment as soon as possible on the stroke unit or, if not possible before discharge, an urgent orthoptic outpatient appointment [9]. In response to these guideline changes, SSNAP added the following questions to the inpatient and community datasets: date screened for visual impairment using a standardised tool and date first assessed by an orthoptist [10]. Therefore, the associated SSNAP indicators are the percentage of applicable patients who have vision screening by discharge and assessed by an orthoptist by discharge or have an orthoptic outpatient appointment scheduled by discharge [10].

Eight years have now passed since the publication of the 2016 National Clinical Guidelines for Stroke. Evidence-based guidelines act as important catalysts for quality improvement [11]. Given the substantial updates of these national guidelines, the purpose of this study was to revisit the provision of orthoptic care for stroke services, and to determine what changes had occurred in service provision since the 2016 guidance changes and the very recent 2023 updates.

## Materials and methods

With the support of the British and Irish Orthoptic Society (BIOS) an email/social media advert was distributed, requesting participation of orthoptists based in the UK and Ireland in a professional survey of stroke service provision. In May 2024, there were 1543 Health and Care Professions Council (HCPC) registered orthoptists in the UK, of which 1179 were BIOS members [12]. These orthoptists worked in 186 departments across the UK and Republic of Ireland.

**Fig. 1:**
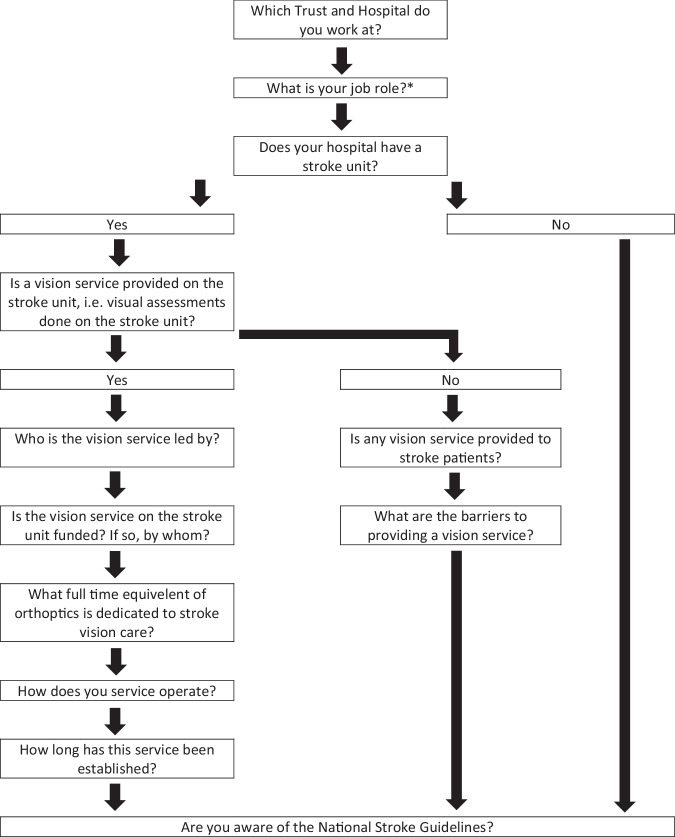
Outline of survey including logic routing.

Specifically, the survey asked the questions outlined in Fig. 1, using logic routing and was delivered via a Qualtrics online platform (Qualtrics, Provo, UT). This study conformed to the Tenets of Helsinki and ethical approval was obtained from the University of Liverpool (Reference: IPH13873). The first page of the survey included the participant information and consent form. Descriptive analysis was undertaken to combine responses in relation to each of the questions. Job role in relation to stroke care and the initials of the person completing the survey were requested to allow responses to be amalgamated by department in cases of multiple responses from the same department. Differences in responses were reconciled by using the job role stated to judge which respondent was most likely to have access to the data being requested.

## Results

### Hospital responses

A total of 206 responses were received. Of these five did not proceed from the initial introduction to the survey and no data was provided. Duplicate responses from the same department were amalgamated to create one response from 125 orthoptic departments and 117 hospital Trusts/Health Boards (organisations responsible for delivering healthcare services to a geographical area or within a specialised area of health, therefore may comprise of a group of hospitals). This equates to a 67.2% response rate from the orthoptic departments across the UK and Republic of Ireland. These responses came from the following geographical spread; England (*n* = 93), Scotland (*n* = 15), Wales (*n* = 7), Northern Ireland (*n* = 5), Ireland (*n* = 1) and other, i.e. crown dependency (*n* = 3).

**Fig. 2:**
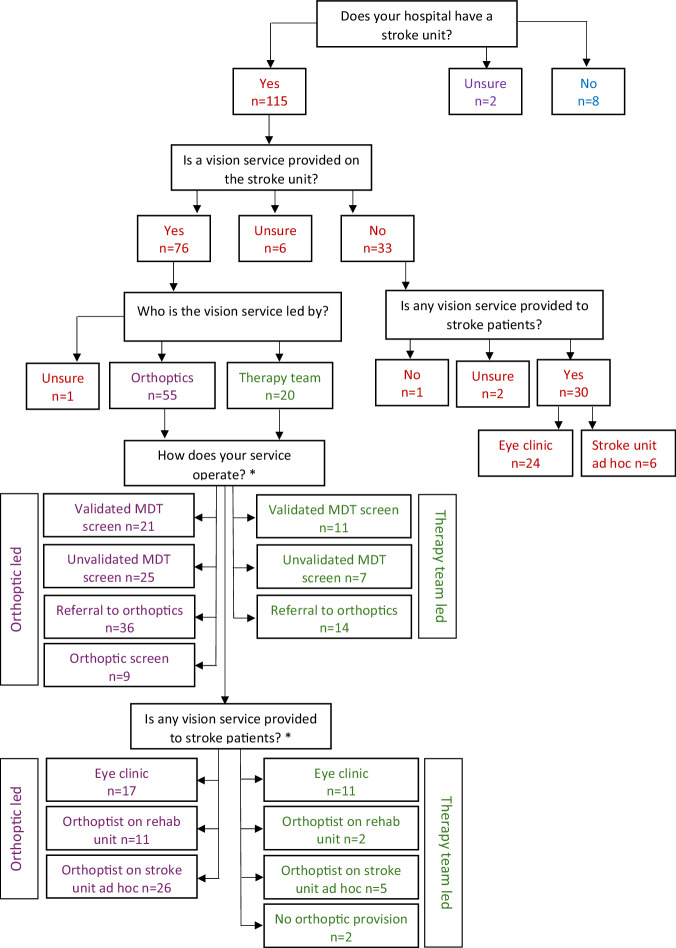
Flow chart of responses regarding provision of stroke vision services (*n* = 125 hospital orthoptic department responses).

A flow chart of responses is outlined in Fig. 2. The majority (92%, *n* = 115) of orthoptic department responses reported having a stroke unit within their hospital. The majority of orthoptic department respondents were aware of the national stroke guidelines (*n* = 117, 93.6%).

A total of 91 (72.8%) responses reported the stroke units linked to their orthoptic department had a mean of 42.5 (SD 21.8) beds. They also reported how these beds are allocated to different levels of stroke care with a mean of 10.2 (SD 12.8) hyperacute beds, 18.5 (SD 12.6) acute beds and 19.0 (SD 14.5) rehabilitation beds.

### Provision of vision services

The provision of a vision service on the stroke unit was reported by approximately two thirds of department responses who had a stroke unit in their hospital (66.1%, *n* = 76) (Fig. 2). Many of these services were led by the orthoptic team (72.4%, *n* = 55) with the remainder led by the therapy team: predominantly occupational therapy (90.0%, *n* = 18). Regardless of which profession led the service there was a high reliance on the multidisciplinary team (MDT) screening (84.2%, *n* = 64) and referral to orthoptics (65.8%, *n* = 50). There was an equal split between validated (50.0%, *n* = 32) and unvalidated (50.0%, *n* = 32) screening tools used by the MDT. Only 11.8% (*n* = 9) of department responses reported blanket screening (i.e. all stroke survivors receiving visual screening) was provided to all stroke admissions by an orthoptist. Most of these departments were in England (88.9%), and frequently the North-West of England (55.6%). There was a high proportion of orthoptic services being provided either in the eye clinic or using ad hoc stroke unit visits (77.6%, *n* = 59). Two (2.6%) department responses reported no orthoptic provision despite screening being provided by the stroke MDT. The mean orthoptist full time equivalent (FTE) dedicated to stroke service provision was 0.43FTE (SD 0.42) and the median 0.25FTE (IQR 0.12–0.65). A Spearman’s rank-order correlation was run to assess the relationship between the orthoptist FTE and number of stroke beds, 68 responses reported both variables, demonstrating a negligible correlation, *r*_*s*_ = 0.184, *p* = 0.133.

Of the 33 departments that reported not providing a regular vision service on the stroke unit in their hospital, the majority (90.9%, *n* = 30) reported providing vision assessments for stroke survivors in either the eye clinic or by visiting the stroke unit on an ad hoc basis.

### Funding support for vision services

**Fig. 3:**
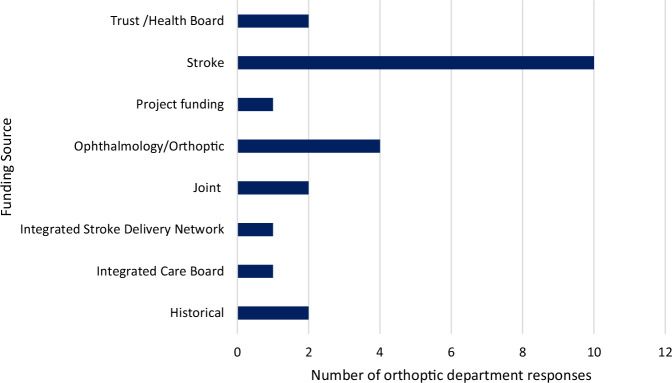
Sources of funding for stroke vision services as described by department responses (*n* = 21).

**Fig. 4:**
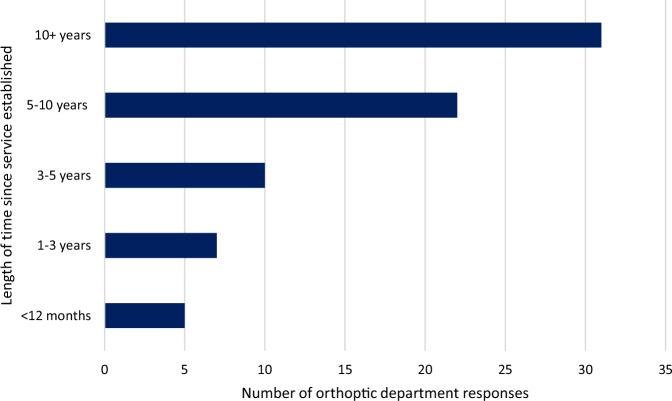
Length of time vision service on the stroke unit has been established (*n* = 76).

Of those that reported a vision service being provided on the stroke unit (*n* = 76), only 21 department responses (27.6%) reported this service to be funded. Sixteen (21.1%) department responses reported not knowing the status of funding for the stroke vision service. A variety of responses were provided in reference to who funded the vision service, which are outlined in Fig. 3. Sources of funding included stroke budget, ophthalmology/orthoptics budget, joint trust/external funds, and commissioners. The majority of services were well established with 69.7% (*n* = 53) being in place for over 5 years. Frequency of amount of time services had been established are outlined in Fig. 4. The mean orthoptist full time equivalent (FTE) dedicated to stroke funded was 0.27FTE (SD 0.44) and the median 0.00FTE (IQR 0.00–0.40). The discrepancy between the actual provision (0.43, SD 0.42) and funded provision was a mean of 0.16FTE (SD 0.20) and median of 0.1FTE (IQR 0.00–0.20).

**Fig. 5:**
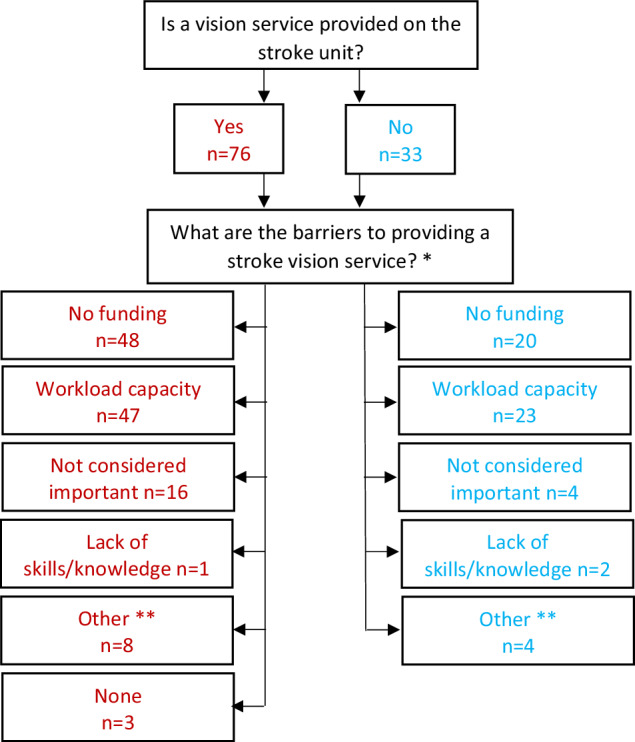
Flow chart of responses regarding barriers to providing stroke vision services (*n* = 109 hospital orthoptic department responses).

The barriers to providing stroke vision services are outlined in Fig. 5. The two most common barriers were lack of funding (62.4%, *n* = 68) and lack of orthoptic capacity (64.2%, *n* = 70) for both departments not currently providing service and those with existing services. Nineteen departments (16.5%) reported that there remained at least one party that did not consider stroke vision services important; ophthalmology (57.9%), stroke team (36.8%) and/or orthoptics (26.3%). Only three departments (2.8%) reported no current barriers to provision of stroke vision services.

## Discussion

This third survey assessing the provision of vision services in stroke care has again shown an increase in the number of departments reporting a vision service on stroke units (66.1%). In 2017, it was reported that 45.5% of departments had a provision of a vision service on the stroke unit with more providing appointments in outpatients. This was an increase from the first survey in 2007 where a high proportion (45%) offered no formal provision of a stroke vision service [6, 7]. Increases in vision service provision likely reflects the inclusion of orthoptists as core members of the MDT in the 2016 National Clinical Guidelines [5]. There was further increase of knowledge of the guidelines amongst respondents, with 93.6% reporting their awareness, up from 85.7% in 2017 and 62% in 2007 [6, 7].

Despite the increase in number of departments reporting a vision service on the stroke unit, there were very few (11.8%) providing blanket orthoptic screening as recommended in the 2023 updated NICE guidelines for stroke rehabilitation [9]. There remains some departments where there is no orthoptic provision for stroke (2.6%), and thus they fail to meet the 2016 and 2023 National Stroke Guideline recommendations [5]. The SSNAP post-acute organisational audit in 2021 reported 29% of post-acute inpatient stroke teams did not have access to orthoptists [13]. There was a high reliance (84.2%) on MDT screening, of which 46.2% reported use of a standardised screening tool (i.e. completed the same way by all users, covering all required domains of the stroke vision screening core outcome set) as recommended in the 2023 National Clinical Guidelines [4]. A core outcome set has been established for vision screening in clinical practice for stroke; the core elements include testing visual acuity, eye alignment, eye movements, visual fields, visual inattention and functional vision [14]. This reliance is supported by the SSNAP national organisational audit with a reduction in access to orthoptists within 5-days from 85% in 2019 to 78% in 2021 [15]. These differences in service provision across stroke units represents a health inequality for stroke survivors, with MDT screening unlikely to detect all visual impairment types especially in cases where not all elements of visual function are being assessed through use of non-standardised screening tools or where there is an over-reliance on symptom reporting [9, 16–20]. This highlights that not all available tools are suitable for vision screening, for example, the National Institute of Health Stroke Scale only assesses for visual field loss and horizontal gaze defects and therefore does not assess all visual function facets [21]. The 2023 NICE guidelines for stroke rehabilitation and 2025 ESO guideline on visual impairment in stroke recommends, in the absence of a specialist visual assessment which would provide improved detection, a validated vision screening tool be used [3]. A validated screening tool will have been assessed in terms of reliability (ability to achieve consistent findings in visual impairment detection), validity (ability that visual impairment can be accurately detected) and sensitivity (probability that visual impairment will be detected if present). Increasingly, multiple guidelines are recommending a specialist visual assessment to identify visual impairment following stroke, which for the UK are orthoptists [3, 9]. In the absence of these services at present, the recommendations now stipulate either a standardised (i.e. completed the same way by all users, covering all required domains of the stroke vision screening core outcome set) or validated (i.e. psychometrically evaluated) screening tools for use by the wider stroke MDT [14]. In addition, the reliance on vision screening by the stroke team followed by referral to orthoptic services creates a time delay for treatment, as reported previously with use of a referral pathway with a first vision assessment (median 19 days) versus pathway using a blanket orthoptic assessment (median 4 days) [2, 22].

The level of orthoptic staffing reported by departments (mean 0.43FTE, median 0.25FTE) falls well below the staffing levels recommended by BIOS and the 2023 National Clinical Guidelines for Stroke [4, 23]. A higher level of staffing does not correlate with larger stroke units. In 2017, the Northern Ireland workforce review identified that an additional 3.4FTE of orthoptic time was required instead of the 2.4FTE already provided to achieve regional equity [24]. An action plan to extend the roll out of orthoptic assessment for all stroke survivors was also set within this report for 2019 to 2029 [24].

This survey indicated a reduction in the number of funded services down to 27.6% from 32.9% in 2017. Funding was reported from a variety of sources across stroke and ophthalmology. The models for paying NHS providers varies across countries and department specialities, including block contracts (payment to deliver a specific service), national tariffs (payment according to activity) and capitation (lump-sum based on number of patients in a target population) [25]. Orthoptists most commonly sit within ophthalmology departments and stroke commonly not factored into the funding model, therefore this work in performed on top of contracted services resulting in ad hoc delivery of care as reported in survey responses. Funding, along with lack of workforce capacity, were common barriers to providing stroke vision services. The latter may be as a direct result of lack of funding or loss of positions. Lack of orthoptic capacity from University-level training of orthoptists was not found to be an issue. Training numbers remain robust and are increasing. The economic evidence section of the NICE guidelines for stroke rehabilitation highlighted that, overall, staff time costs associated with a routine orthoptic assessment on the stroke unit would be lower compared to MDT vision screening followed by orthoptic referral [9]. Cost savings therefore could be made through provision of orthoptic assessments on acute stroke units. Orthoptists are typically on the same salary scale as occupational therapists who most frequently provide the MDT vision screening. Stroke survivors with visual impairment would be identified and receive treatment quicker when seen by orthoptists negating referral requirements (and associated costs) to ophthalmology. This would also facilitate a more efficient use of the MDT skill mix [9]. In addition, there is the potential for downstream savings associated with prevention of falls and driving accidents associated with visual impairment [9]. Early identification and treatment of visual impairment can also improve the experience of stroke survivors, improve engagement with MDT rehabilitation, and reduce impact on quality of life; equally important for stroke survivors with pre-existing visual impairment [8, 26, 27].

Studies in Canada and Norway highlight that other countries are attempting to implement early vision assessments within stroke units [28, 29]. The Norwegian study identified barriers to implementation of these services, including lack of ownership of vision assessments, lack of interdisciplinary collaboration and time constraints [28]. Other countries are using alternative methods of vision assessment in the absence of orthoptists, such as tele-consultations with ophthalmologists with a focus on retinal artery occlusion (ocular stroke) rather than visual impairment associated with cerebral stroke [30]. These studies highlight that vision assessment across different countries remains a challenge. However, in the UK and Ireland there is an orthoptic workforce which can be utilised with both clinical and cost effectiveness within stroke care.

A limitation of this study is that not all orthoptic departments responded to the survey. It is possible that departments with better services were more likely to respond, potentially overestimating the provision of services. Alternatively, weaker services may have been more likely to respond in order to highlight their need for support, potentially underestimating the provision of services. Only one response was received from the Republic of Ireland, it is only since 2023 that the UK and Ireland have had guideline which has applied across the five nations [4]. This survey also only collected the perspective of the orthoptic profession although this was essential to gather information on the provision of orthoptic services on stroke units. Incorporating multidisciplinary viewpoints (e.g., stroke team, commissioners) could broaden insight. This study was conducted in the UK and Ireland and therefore is not generalisable to countries who do not have routine access to orthoptists. However, the question could be asked about which professions are providing vision screening post-stroke and specialist eye services in other countries given the occurrence of visual impairment post-stroke is known to be similar to the UK [29].

## Conclusions

Since 2017 there has been an increase of vision screening provided on stroke units but with a reduction in funded orthoptic services on these units. Despite the increase in service there remain areas with no/poor provision of post-stroke vision care representing a health inequality. The National Clinical Guidelines for Stroke and the NICE Stroke Rehabilitation Guidelines were updated in 2023, recommending people with stroke should be screened for visual changes, or have specialist orthoptist assessment, as soon as possible, respectively. SSNAP recently (2024) implemented changes to the stroke data collection to include the date of screening for visual impairment and/or orthoptic assessment within both the inpatient and community datasets. The survey results presented here provide a baseline by which to assess change following these strengthened recommendations/guidelines.

## Summary

### What was known before

- A survey of UK orthoptic stroke services in 2007 found that 45% of stroke services did not provide any formal vision assessment. In 2017 there remained a lack of orthoptists providing a service specifically on stroke units (48%) and vision care was being delivered in outpatient clinics for many stroke survivors. Three new guidelines relating to post-stroke vision were published between 2023–2025, and added to a mandatory national audit programme.

### What this study adds

- There has been an increase of vision screening provided on stroke units but with a reduction in funded orthoptic services on these units. Despite the increase in service there remain areas with no/poor provision of post-stroke vision care representing a health inequality. The survey results presented here provide a baseline by which to assess change following these strengthened recommendations/guidelines.

## Data Availability

The dataset generated during and analysed during the current study are available from the corresponding author on reasonable request.
